# Report on the 12th international workshop on the CCN family of genes, Oslo, June 20–23, 2024

**DOI:** 10.1002/ccs3.12049

**Published:** 2024-08-13

**Authors:** Håvard Attramadal, Ralf Weiskirchen, Bernard Perbal

**Affiliations:** ^1^ Faculty of Medicine Institute of Clinical Medicine University of Oslo Oslo Norway; ^2^ Institute for Surgical Research Oslo University Hospital Rikshospitalet Oslo Norway; ^3^ Institute of Molecular Pathobiochemistry Experimental Gene Therapy and Clinical Chemistry (IFMPEGKC) RWTH University Hospital Aachen Aachen Germany; ^4^ International CCN Society Nice France

**Keywords:** cancer, CCN1, CCN2, CCN3, CCN4, CCN5, CCN6, cellular signaling and communication, development and homeostasis, fibrosis extracellular matrix, international CCN workshop, matricellular proteins, vascular development and pathophysiology

## Abstract

The 12th international workshop on the CCN family of genes took place at the Scandic Holmenkollen Park Hotel in Oslo, Norway from June 20–23, 2024. In 2024, it was the second time, following the Nice meeting in 2022, that the scientific topics were expanded to include additional cellular signaling and communication pathways of interest to the CCN Society members, as suggested by Bernard Perbal in 2019. The 12th international CCN workshop, organized by Håvard Attramadal and Vivi T. Monsen, along with co‐organizers Bernard and Annick Perbal, was given the subtitle “Cell‐matrix Communication and Functions in Health and Disease” to encompass the broader scope of this meeting. The five scientific sessions covered various topics: Extracellular Matrix Proteins in Cell Communication and Signaling (Chaired by Brahim Chaqour and Vivi T. Monsen), Vascular Development and Pathophysiology (Chaired by Lester F. Lau and Håvard Attramadal), Mechanisms of Diseases (Chaired by George Bou‐Gharios and Satoshi Kubota), Tissue Development and Homeostasis (Chaired by Blandine Poulet and Bernard Perbal), and Mechanisms of Disease: Cancer and the Matrix (Chaired by Stephen M. Twigg and Raymond B. Birge). The 2024 ICCNS Award was presented to Katia Scotlandi during the last session (Chaired by Bernard Perbal) before Håvard Attramadal presented the conclusion of the workshop.

## Graphical abstract

Schematic representation of CCN proteins involvement in cell communication and signaling.

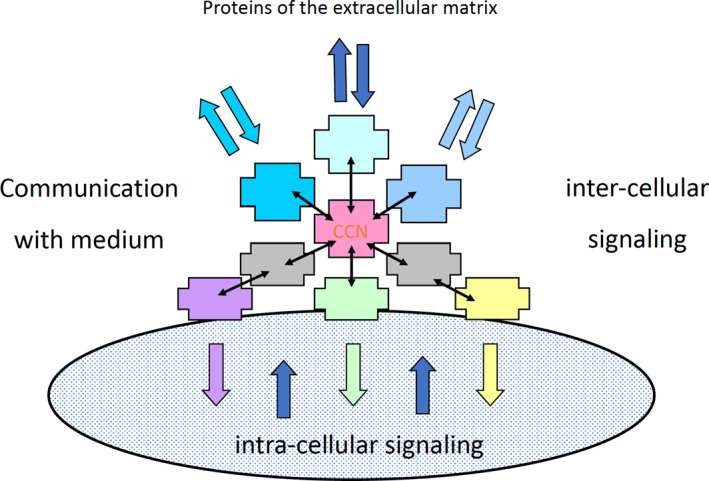

## 1 JCCS EDITORIAL BOARD MEETING

**Annick Perbal**, Administrative Coordinator from the Journal of Cell Communication and Signaling organized a meeting in which members of the Editorial Board were invited to comment on the achievements of the ICCNS journal, presented by its Editor in Chief, **Bernard Perbal**. A total of 18 Editors participated in the discussion.

The first part of the report consisted of a review of the 2023 metrics of JCCS, which was still published by Springer Nature until the transition to Wiley occurred. The values presented showed a remarkable 46% increase (compared to 34% in the previous year) in the number of JCCS downloads, jumping from 143,744 in 2022 to 210,196 in 2023. As discussed in a JCCS Editorial published by Bernard Perbal at the end of 2023, this increase is seen as a sign of readership expansion, reflecting a growing interest from the scientific community in the content of JCCS manuscripts. A comparison of the 2023 JCCS metrics (IF, Cite score, SNIP, and H5 Index) was also presented and discussed by the Editors during this meeting, all indicating that JCCS was in good shape.

The second part of the discussion focused on the metrics of JCCS over the past 5 months since the transition to Wiley was completed. The values provided a general trend confirming that the expected rebirth of JCCS as a Gold Open Access publication was a positive move, with a 19% acceptance rate at this time. Other data remained confidential. Aspects regarding the future development of JCCS were also discussed, showing that the Editors were satisfied with the new workflow that was presented in detail.

As a token of appreciation for their work, the Editors were invited to a special lunch offered by the ICCNS.

## 2 ICCNS AWARD AND SCIENTIFIC SESSIONS

The International CCN Society was proud to award **Katia Scotlandi** from the IRCCS Rizzoli Institute in Bologna, Italy for her outstanding contributions to pediatric cancer research. Her scientific contribution was not only in a field where she unraveled new mechanisms of carcinogenesis and the role of epigenetic mechanisms governing the instability of the tumor phenotype of cancer cells. Katia Scotlandi has been and still is a very active researcher and mentor. Among the many researchers that she trained was the President of the ICCNS, with whom she shared her knowledge and bright ideas about the participation of CCN3 in assessing the metastatic potential of musculoskeletal tumors. In these models, the production of isoforms containing several combinations of the four constitutive modules was found to match developmental stages, both in normal and pathological conditions. This productive collaboration gave rise to several publications. Only many years later, similar observations were made in melanoma cells and in neural development.

The Executive ICCNS Committee is sincerely honored to celebrate the professional success and enthusiasm of Katia Scotlandi. In her fascinating and elegant presentation, Katia Scotlandi showed how a tumor with a similar diploid karyotype and only one well‐recognized oncogenic driver (the chimera EWS::FLI resulting from a reciprocal chromosomal translocation) may indeed be very complex. Katia Scotlandi has spent most of her scientific career studying Ewing sarcoma, a rare pediatric bone tumor. Ewing sarcoma originates from a mesenchymal stem cell that is blocked in its differentiation process by the occurrence of a chromosomal translocation that forms a fusion product acting as an aberrant transcriptional factor. The chimera acts as the oncogenic driver of Ewing sarcoma, which through its binding to DNA, completely changes the genetic landscape of the transformed cell. Despite being necessary, the presence of the chimera is not sufficient for a fully transformed phenotype, and other players contribute to sustaining Ewing sarcoma malignancy, such as CD99 and the RNA binding protein IGF2BP3. Katia Scotlandi illustrated the mechanisms of action of IGF2BP3 in cancer, particularly in Ewing sarcoma, highlighting its relevance as a prognostic biomarker and therapeutic target. She also pointed out how a new targeting approach (protein targeting degradation) may be valuable in inhibiting such an oncogenic protein.

## 3 WORKSHOP OPENING

The 12th workshop opened with a nice welcome from the local organizer Havard Attramadal who acknowledged sponsorships from the University of Oslo: Life Science, the Oslo University Hospital and the International CCN Society. Thanks were also addressed to the members of the Scientific Advisory Board for their help and to all those who came to participate in the Oslo Workshop.

In his Scientific Introduction entitled “*Where do we come from and where are we going*,” **Bernard Perbal** proposed a “*fascinating journey in time*,” including a review of both the universe origin and its expansion, confirmed by the James Web Space telescope recent data, and abiotic chemistry reactions leading to the RNA World and origins of life. The fusion of RNA metabolism and RNA genome occurring in early lipid bilayer vesicles paved the way to LUCA (the Last Universal Common Ancestor) and to the three domains of life (Archea, Bacteria, and Eukarya). Shuffling of genetic information resulted in the combination of functional domains (IGFBP, VWC, and TSP1) represented in 500 million‐year old living species, while the CT domain seems to appear about 250 million‐year ago in *Drosophila* combination with VWC and TSP1.

Further to these considerations, Bernard Perbal quickly reviewed the salient features of CCN proteins with an emphasis on the wide array of their biological functions. The spatio‐temporal model that Bernard Perbal proposed several years ago to account for the variety of CCN proteins biological activities is based on the combinatorial events permitted by the bioavailability of CCN proteins and ligands with which they can interact in various tissues at different times under normal and pathological conditions.

Bernard Perbal ended up his talk with a few examples of CCN proteins biological functions and localizations that are potentially of great interest for future collaborative explorations, paving the road to a better understanding of this tetramodular family of proteins and potential biomedical applications.

**Annick Perbal** provided a brief presentation summarizing the past, present, and future activities of ICCNS. She first mentioned the key principles that have guided our successful workshops since 2000, which are as follows. (i) Equal presentation time for all presenters, (ii) no fully‐invited speakers except for educational sessions, (iii) sponsorship of special events by ICCNS, (iv) encouraging all presenters to stay for the entire meeting to promote interactions between senior and junior members, (v) reactivation of successful educational sessions, and (vi) recognition of colleagues who have accepted the ICCNS‐SPRINGER awards, with hopes that our new publisher will support future nominations. Annick Perbal noted that, despite the growing interest in supporting our workshops within the scientific community, there has been a lack of sponsorship. This has not deterred increased attendance and participation in our meetings. A call for memberships received a positive response from the audience. Requests for invoices can be sent to ccnsociety@yahoo.com.

The current composition of ICCNS can be found on the CCN society website. It will be adjusted to align with the development of the ARBIOCOM project, and elections will be held in the upcoming year.

Finally, on behalf of the ICCNS, Annick Perbal expressed gratitude to Joon‐Il Jun for agreeing to be the local organizer of the next CCN workshop in Chicago.

## 4 SCIENTIFIC SESSIONS

### 4.1 Session I: ECM proteins in cell communication and signaling

**Donald Gullberg**, from the University of Bergen, Norway, presented a talk on collagen receptors, focusing mainly on the “fibrotic niche marker integrin α11β1.” This integrin was first described and characterized in his laboratory by Velling et al. in 1999. To understand the biology of collagen‐binding integrins, Donald Gullberg discussed integrin signaling, the availability of integrin‐binding sites in collagen fibrils in newly formed versus mature fibrils, and the recent discovery of fibroblast heterogeneity. The role of integrin α11β1 in tissue and tumor fibrosis was highlighted based on recent publications showing its expression in myofibroblastic cancer‐associated fibroblasts and pro‐fibrotic myofibroblasts (Zeltz et al., *J Cell Commun Signal* 2022; 16:649‐60). Donald Gullberg concluded his seminar by sharing some ongoing projects in his laboratory, including epitope mapping of integrin α11 antibodies, determining the roles of individual amino acids in the cytoplasmic tail of integrin α11, and mentioning the ongoing project of generating a humanized mouse model of integrin α11 in collaboration with Biocytogen. This new model is expected to be valuable in testing anti‐fibrotic reagents in tissue and tumor fibrosis models.

**Raymond B. Birge**, from Rutgers New Jersey Medical School, USA, gave a lecture on the recent discovery of the mechanisms of efferocytosis of apoptotic cells and the suppression of immune responses by engaging a series of phosphatidylserine receptors called TIM (T cell, Immunoglobulin, and Mucin) and TAM (Tyro3, Axl, and Mertk) receptors. He also discussed strategies to target the phosphatidylserine/phosphatidylserine receptor axis in cancer in order to abrogate immune evasion, including therapeutic antibodies targeting phosphatidylserine or MERTK for cancer immunotherapy.

**Joon‐Il Jun**, from the University of Illinois at Chicago, USA, presented novel functions of CCN1 in the regulation of intestinal epithelial regeneration after injury. Dr Jun and his colleagues showed that CCN1 is a key regulator of intestinal regeneration during homeostasis through the coordinated regulation of intestinal stem cell (ISC) proliferation and differentiation of the various cellular elements of the intestinal epithelium. Importantly, the research group also provided evidence that CCN1 may repair the intestinal epithelium following severe injury with a loss of ISCs by promoting dedifferentiation of lineage‐committed cells into ISCs, allowing for the regeneration of the intestinal epithelium.

**Vanja Pekovic‐Vaughan**, from the University of Liverpool, UK, presented new data implicating the circadian/NRF2 signaling axis in the regulation of extracellular matrix gene expression. Her group found a robust diurnal variation in tissue repair after injury in vivo controlled by the circadian/NRF2 signaling pathway. Interestingly, she reported that the decreased diurnal rhythmicity of NRF2 levels as individual age was linked to a decline in circadian control of tissue healing. These findings could have significant implications for the development of therapeutic approaches aimed at addressing age‐related fibrosis.

**Taihao Quan**, from the University of Michigan, USA, demonstrated that matricellular CCN1 enhances skin aging by causing the fragmentation and disorganization of collagen fibrils in the dermis, as well as creating a pro‐inflammatory microenvironment. Furthermore, mice with dermal fibroblast‐specific overexpression and secretion of CCN1 showed an increased susceptibility to keratinocyte cancer in mouse models of skin carcinogenesis, including the cutaneous two‐stage chemical carcinogenesis model and the inducible HRas tumor model. Interestingly, the aging of skin induced by CCN1 was a prerequisite for the increased propensity of tumor formation in mice overexpressing CCN1. Consistent with these findings, mice with fibroblast‐specific knockout of CCN1 exhibited a decreased propensity for skin tumor formation.

### 4.2 Session II: Vascular development and pathophysiology

Session II on vascular development and pathophysiology featured several talks presenting novel data on the key roles of CCN proteins (CCN1 and CCN2) in neoangiogenesis, vascular integrity, and the pathophysiology of arterial aneurysms. **Anna Zampetaki**, from King's College London, UK, presented data showing that CCN2 is necessary for the vascular development of induced pluripotent stem cells. CCN2 not only promoted the differentiation of endothelial cells and simple tube formation from induced pluripotent stem cells but also perivascular cells. Additionally, CCN2 played a role in metabolic adaptation to the hypoxic environment that precedes the formation of a patent vascular circulation.

Using atomic force microscopy, **Brahim Chaqour**, from the University of Pennsylvania, USA, presented data showing that CCN2 increased the mechanical rigidity of the retinal vasculature and exacerbated retinal neovascularization in mice subjected to oxygen‐induced retinopathy. Indeed, endothelial‐specific overexpression of CCN2 in mice subjected to oxygen‐induced retinopathy led to retinal neovascularization and the formation of star‐shaped neovascular tufts with increased stiffness compared to neovascular tufts of wild‐type mice. The data suggest that CCN2 may be implicated in the microvascular diseases associated with diabetes, hyperlipidemia, and ischemia.

**Fan E. Mo** from the National Cheng Kung University of Taiwan presented new evidence indicating that CCN1 plays a crucial role in the formation of aortic aneurysms. The authors discovered that CCN1 expression was stimulated in vascular smooth muscle cells and infiltrating macrophages, and was associated with heightened levels of inflammation and oxidative stress in the lesion areas of the CaCl_2_‐induced model of aortic aneurysm formation. By utilizing genetically modified mice carrying a mutant knock‐in allele of CCN1 that was defective in binding to integrin α_6_β_1_, the researchers observed that the structure of the aortic wall remained remarkably intact following CaCl_2_ treatment. Therefore, Fan Mo concluded that CCN1 could potentially serve as a new therapeutic target for aortic aneurysms.

In a subsequent presentation, **Zhiyong Lin**, from Emory University in Atlanta, USA, shared evidence suggesting that CCN2 secreted from vascular smooth muscle cells may actually protect against the development of aortic aneurysms. His team demonstrated that hypercholesterolemic mice with smooth muscle‐restricted deletion of CCN2, when infused with angiotensin II, developed aortic aneurysms in the infrarenal aorta, whereas wild‐type control mice were resistant to angiotensin II‐induced aneurysm formation. Interestingly, the authors observed that CCN2 deficiency led to a phenotypic switch in smooth muscle cells that seemed to contribute to aneurysm formation. The differing roles of CCN1 and CCN2 in the pathophysiology of aneurysm formation are intriguing, but require further investigation as the Mo group and the Lin group used different disease models. Both CCN1 and CCN2 have been implicated in the pathogenesis of atherosclerosis, which is a key factor in aneurysm formation.

**Mei‐Zhen Cui**, from the University of Texas Permian Basin, USA, presented data showing that lysophosphatidic acid, a plasma membrane derivative that accumulates significantly within atherosclerotic plaques induces CCN2 expression via LPA receptor 1 (LPA1) in vascular smooth muscle cells. Furthermore, M‐Z. Cui demonstrated that CCN2 enhances the proliferation of vascular smooth muscle cells, significantly contributing to vascular plaque formation.

### 4.3 Session III: Mechanism of diseases: Fibrosis and the matrix

**Sylvie Ricard‐Blum**, from the University of Lyon, France, presented data on the structure–function relationships of glycosaminoglycans and syndecans. These transmembrane proteoglycans are abundant molecules associated with the cell surface and extracellular matrix, playing a variety of important biological roles. She discussed how alterations of the polysaccharide side chains attached to the core protein may impact function, including ligand binding selectivity. Glycosaminoglycans play a crucial role in controlling the availability and function of a diverse array of matricellular proteins. In particular, syndecans are upregulated in a range of inflammatory conditions.

**Andreas Romaine**, from the University of Oslo, presented data on cardiac fibrosis in aging rats. He reported that cardiac fibrosis precedes the development of diastolic dysfunction in aging rats and is associated with the specific upregulation of integrin α11. This integrin has an affinity for collagen, acting as a mechanic link between the cytoskeleton and extracellular ligands. It is involved in the control of cell proliferation, cell migration, and cell differentiation. As an expert in studying integrin and proteoglycan biology for the activation of cardiac fibroblasts, Andreas Romaine's work suggests that integrin α11 might be a potential therapeutic target for cardiac fibrosis.

**Morten A. Karsdal**, from Nordic Bioscience, Inc. and the University of Southern Denmark, presented data on several new candidate biomarkers for fibrosis. He highlighted the two main mechanisms of increased collagen deposition in fibrosis: increased synthesis and decreased degradation of collagens. Following this, he discussed data on candidate biomarkers for collagen synthesis and degradation. One potential biomarker he mentioned was Pro‐C3, a plasma biomarker derived from the propeptide of collagen III, which could indicate increased collagen synthesis in liver fibrosis related to steatohepatitis. Additionally, he discussed data on endotrophin, a degradation product of collagen VI‐α3, currently being clinically tested as a biomarker for cardiac dysfunction in diastolic heart failure.

**Vivi T. Monsen**, from the University of Oslo, presented data demonstrating that the carboxyl‐terminal thrombospondin 1 (TSP1) homology domain of CCN5 is the bioactive entity mediating the signaling functions of CCN5. This includes the suppression of TGF‐β signaling, leading to the reversal of epithelial‐to‐mesenchymal transition in pulmonary fibroblasts. Moreover, cell migration and gap closure following scratch wound in these cells induced by CCN2 were significantly inhibited by TSP1 of CCN5. These findings support the anti‐fibrotic role of CCN5 and demonstrate that the TSP1 domain of CCN5 is sufficient to induce a range of reported functions achieved by full‐length CCN5.

**Ole J. Kaasbøll**, the Chief Scientific Officer of Tribune Therapeutics, Inc. in Oslo, Norway, presented the company's work on developing novel biological therapeutics that target CCN proteins in diseases associated with fibrosis. Tribune Therapeutics is a recently established biotechnology company focusing on developing new medicines that efficiently target multiple drivers of fibrosis to prevent scar formation. The company is particularly interested in key members of the CCN family, such as CCN2, which play a central role in the pro‐fibrotic pathway.

**Danqing Min**, from the University of Sydney, highlighted the biological activity of CD163^+^ monocytes in the pathogenesis of diabetes with a special emphasis on diabetic retinopathy. CD163 is a marker of cells from the monocyte/macrophage lineage that also functions as an innate immune sensor for bacteria and as a high affinity scavenger receptor for the hemoglobin–haptoglobin complex. In the study presented, RNA sequencing and mass cytometry were conducted in circulating CD163^+^ monocytes in adults with long‐duration diabetes, with or without diabetic complications. RNA sequencing identified a total of 885 upregulated and 190 downregulated genes in patients with diabetic complications compared to healthy individuals. Specifically, genes that regulate the centrosome cycle were differentially expressed. PLK2, CENATAC, RBM14, CCNL2, KAT2A, CCNL1, XRCC3, CEP295NL, CEP131, CDK1B, and CDK11A were enriched in diabetic monocytes. Additionally, miR27A, miR3648‐1, and miR23A were the most upregulated genes, while CD200R1 was the most downregulated gene in patients with diabetic complications. Moreover, CD163^+^ monocytes taken from diabetic patients exhibited a low proportion of the recruitment markers CCR5, CD11b, CD11c, and CD31, and the immune regulation markers CD39 and CD86. A detailed gene‐protein network analysis identified TLR4 and CD11b as “hub‐nodes” that are suppressed in CD163+ monocytes from diabetic patients. These data suggest that diabetic complications are associated with altered expression of centrosome cycle genes and miRNAs that might interfere with the anti‐inflammatory activity of CD163^+^ monocytes, thereby limiting their potential to resolve inflammation during diabetes.

Another presentation was given by **Stephen M. Twigg**, also from the University of Sydney, which continued the discussion on diabetes and its complications. During his talk, he provided an update on CCNs as markers and mediators in diabetes and its complications, with a specific focus on NASH and lifestyle factors. Stephen Twigg highlighted liver phenotypes, the impact of lifestyle, and the biological activity of CCN proteins in these processes. His research, built upon previous findings, shows that liver CCNs are dysregulated in mice that are fed a high‐fat diet combined with diabetes (FaD), whereas a high‐fat diet alone did not affect hepatic CCN expression. Additionally, Stephen Twigg discovered that blocking CCN2 bioactivity with a systemic anti‐CCN2 antibody could prevent NASH pathogenesis. In his new data, Stephen Twigg disrupted CCN1 or CCN2 in hepatocytes using the Cre‐LoxP system. When exposed to the FaD model, both strains developed a liver phenotype. However, mice with disrupted hepatic CCN1 also displayed a systemic metabolic phenotype, similar to human metabolic syndrome, commonly seen in patients with type 2 diabetes. Conversely, mice lacking hepatic expression of CCN2 showed reduced inflammation and fibrosis. Moreover, Stephen Twigg showed that high‐intensity interval training could prevent fibrosis in the FaD model, although it did not normalize the expression of CCN proteins. These findings suggest that coordinated regulation of hepatocyte CCN1 and CCN2 could prevent NASH fibrosis in type 2 diabetes with high‐fat feeding, but they do not explain the positive effects of exercise in NASH. Overall, this research supports the need for human studies on molecular targeting of CCNs in NASH.

### 4.4 Session IV: Tissue development and homeostasis

**Milos Marinkovic**, from the University of Texas Health Science Center at San Antonio, presented a research on the role of CCN1 in aging bone niches. Previous studies by Milos Marinkovic have demonstrated that the expression of CCN1 is significantly reduced in human and mice bone marrow stromal cells (BMSCs) as they age. In his presentation, he shared data from an in vitro CCN1‐deficient extracellular matrix (ECM). The ECM model was developed using primary aging human BMSCs and an immortalized human BMSC line, HS‐5, which were treated with a CRISPR‐SpyCas9 system to delete CCN1. In his study, Milos Marinkovic used nanocarriers loaded with recombinant CCN1 to restore the loss of CCN1. He discovered that knocking down CCN1 in young ECM eliminated the responsiveness of cultured progenitors to both BMP‐2 and IGF‐1. However, aging ECM, enriched with exogenous CCN1, significantly restored responsiveness to both growth factors. Furthermore, genetic ablation of CCN1 eliminated BMP‐2 responsiveness, suggesting that CCN1 deficiency independently reduces osteogenic signaling in the aging bone marrow matrix. These findings support the notion that CCN1 is a critical ECM component that maintains essential functions in the bone marrow and influences the responsiveness of bone marrow progenitors to osteogenic growth factors.

**Satoshi Kubota**, from Okayama University Graduate School of Medicine, presented data on an inverse genetic approach that enables the reprogramming of somatic cells back to induced pluripotent cells (iPSCs). To demonstrate the effectiveness of his genetic approach, he utilized normal human chondrocytes in which the expression of Yamanaka factors was induced. The resulting differences were analyzed through a time‐course single‐cell transcriptomic analysis, revealing that a limited population of chondrocytes reduced expression of SOX9 and reverted to NANOG‐positive iPSCs. The biological role of SOX9 in maintaining a differentiated chondrocyte phenotype was further confirmed through IPS‐interference experiments. Interestingly, several off‐target cells were identified that exhibited transient induction of CCN1, CCN2, and CCN4, which are believed to influence the differentiation fate of these cells. It is evident that the novel inverse genetic approach presented by Satoshi Kubota may be suitable for identifying master regulators such as SOX9 in any somatic cell in a retrospective manner, and that transient induction of ECM proteins such as CCNs could be effective in treating not only cartilage degenerative diseases.

**Masaharu Takigawa**, who also works at Okayama University Graduate School of Medicine, presented data on the role of polyamines and CCN2 in mediating the chondroprotective action of S‐adenosylmethionine (SAM). The background of this study includes previous findings that CCN2 promotes endochondral ossification, regeneration of injured articular cartilage, and exhibits chondroprotective effects during aging. Polyamines, on the other hand, have the ability to induce chondrocyte differentiation. Masaharu Takigawa demonstrated that SAM produces chondroprotective effects by increasing the expression of proteoglycans, aggrecan, collagen type II, CCN, chondroitin sulfate biosynthetic enzymes, and SOX9. Conversely, blocking methionine adenosyltransferase 2A, which catalyzes intracellular SAM biosynthesis, inhibited the biological effects on chondrocytes. Masaharu Takigawa also showed through alcian blue staining and RT‐qPCR that inhibiting polyamine synthesis with *α*‐difluormethylornithine reduced the effects of SAM on aggrecan expression. In conclusion, the data suggest that stimulating polyamine synthesis and the gene expression of chondrogenic differentiation factors, such as CCN2, are the mechanisms underlying the action of SAM on chondrocytes.

**Nai‐Yuan Cheng**, from the University of Illinois, Chicago, highlighted the mechanisms by which CCN1 regulates intestinal regenerative responses. The starting point is the understanding that during injury, self‐renewal, and differentiation, ISCs differentiate into mature epithelial cells and that severe injury requires the transient reprogramming of subsets of differentiated cells into a fetal epithelium‐like state. Nai‐Yuan Cheng investigated the underlying damage‐associated extracellular signals and the downstream pathways that drive the reprogramming process in crypt epithelial cells of mice subjected to abdominal irradiation injury. He found that mice lacking CCN1 failed to repair damaged epithelium and exhibited higher mortality compared to wild‐type mice. The failure was associated with reduced activation of NOTCH and YAP, as well as fetal epithelium‐associated gene signatures. In line with this, single‐cell RNA sequencing analysis confirmed that CCN1 is simultaneously expressed with fetal marker genes, suggesting that CCN1 plays a crucial role in intestinal regeneration.

**Li‐Jen Lee**, from the National Taiwan University, investigated the function of CCN2 in the brain by using a forebrain‐specific CCN2 conditional knockout mouse strain, with a special focus on behavioral changes. Interestingly, male mice lacking CCN2 exhibited normal locomotion, sensorimotor gating, and social behaviors as demonstrated by a variety of different test systems. However, these mice showed signs of anxiety and elevated aggression as assessed in the resident–intruder task. Molecularly, the mice exhibited increased levels of c‐*fos* in aggression‐related brain regions compared to controls when subjected to aggression testing. In contrast, c‐*fos* quantities were lower in the prefrontal cortex, suggesting that prefrontal neurons are not fully activated during the test used for assessing aggressive behavior. Although the precise function of CCN2 in impacting aggression is not understood, Li‐Jen Lee's presentation showed that the CCN2 protein might be an attractive therapeutic target for treating subjects with neuropsychiatric disorders experiencing anxiety or depression‐related symptoms.

**Danqing Min** presented data on differences in circulating levels of matrix metalloproteinases (MMPs) and tissue inhibitors (TIMPs) in overweight or obese individuals with normal glucose levels, as well as those with diabetes and prediabetes. In her study, she measured the quantities of MMP‐1, MMP‐2, MMP‐3, MMP‐7, MMP‐9, TIMP‐1, and TIMP‐2 in plasma samples. Danqing Min reported that MMP‐7 and MMP‐9 levels were significantly higher in patients with prediabetes, while MMP‐2 was lower in patients with diabetes. TIMP‐1 was found to be higher in both patients with prediabetes and patients with diabetes. Interestingly, exercise decreased MMP‐7 and MMP‐9 levels in patients with prediabetes, while MMP‐2 was lowered in subjects with normal glucose levels and prediabetes. Furthermore, TIMP‐1 increased after exercise in individuals with normal glucose levels, while TIMP‐2 increased in all groups. These measurements demonstrate that the MMP/TIMP system can be modulated by exercise and is dysregulated in overweight and obese individuals with prediabetes and diabetes, suggesting a potential role of these proteins in different stages of inflammation and fibrosis.

### 4.5 Session V: Mechanisms of Disease: Cancer and the matrix

**Bhudev C. Das** presented new data on strategies for targeting invasive cervical cancer, in which human papillomavirus (HPV) oncogenes E6 and E7 contribute to the transformation of epithelial cells. Bhudev Das showed that cervical cancer stem‐like cells from HPV‐negative and HPV‐positive cervical cancer cell lines can form spheroids in culture and possess pluripotency, quiescence, self‐renewal properties, and highly tumorigenic properties in nude mice due to high expression of E6 and Hes1. Bhudev Das showed that knocking down of E6 and Hes1 resulted in the abolition of spheroid formation, suggesting a loss of self‐renewing ability. He further discussed the role of the AP‐1 family member FRA‐1, which has become an attractive target for anticancer interventions. Bhudev Das demonstrated that the phenolic pigment curcumin in combination with UV irradiation triggers the expression of FRA‐1, leading to chemo‐radio sensitization of the cervical cancer stem cells, which are most critical in the formation of drug resistance, tumor relapse, and aggressive metastasis. Bhudev Das presented convincing data that a novel triple bioconjugate, termed CUR‐FA‐DOX, is an effective therapeutic mean. It effectively targets tumor cells, inhibits their growth, proliferation, invasion, migration, and stemness of cervical cancer stem cells, and further restores AP‐1/FRA‐1 expression, thereby overcoming drug resistance and sensitizing the cells to chemo‐radiotherapy.

**Celina G. Kleer**, from the Department of Pathology at the University of Michigan, focused her talk on the molecular pathways through which CCN6 reduces breast cancer progression. Previous work has shown that the deletion of CCN6 induces spindle metaplastic breast carcinomas with increased WNT/β‐catenin signaling. In her new study, she modulated CCN6 expression in patient tissues, MDA‐MB‐231 cells, CCN6‐null primary tumor cells, and non‐tumorigenic HME cells using ectopic expression, CCN6‐enriched media, recombinant CCN6, and shRNA strategies. These studies revealed that CCN6 protein interacts with the WNT receptor FZD8 and co‐receptor LRP6 on metaplastic breast carcinoma cells, thereby antagonizing WNT ligand‐mediated activation of *β*‐catenin/TCF transcription. This results in lower activity of the EZH2 methyltransferase, decreased H3 lysine methylation, and deregulation of specific gene targets. This suggests that restoration of CCN6 or inhibition of *β*‐catenin or EZH2 could have therapeutic benefits for patients suffering from breast cancer tumors.

In one of the most impressive presentations, **Björn Högberg** (Department of Medical Biochemistry and Biophysics, Karolinska Institutet, Stockholm, Sweden) reported on recent developments in DNA‐origami nanotechnology. This methodology allows for the design of nanoscale shapes with unprecedented resolution. In several examples generated in his laboratory, he showed that these origamis can be chemically conjugated to proteins, creating molecularly precise patterns and structures. He demonstrated that DNA nanostructures decorated with ligands involved in cell–cell signaling are effective in deciphering and studying membrane‐bound receptor–ligand signaling systems. As an example, Björn Högberg discussed a recent project in which he used well‐defined molecularly precise patterns of Jag1 ligands capable of activating the Notch pathway from a solution phase without the need for a force activation mechanism. Interestingly, increasing the number of Jag1 ligands on the nanoparticle resulted in increased activation efficiency. This suggests that DNA origami nanostructures are not only useful for addressing biochemical aspects of ligand–receptor interactions, but can also be artificially programmed to enhance the activity of a specific pathway. In a recent study published on July 1 in Nature Nanotechnology, Björn Högberg's team showcased the use of “a stimuli‐responsive robotic switch nanodevice” (Wang et al., *Nat Nanotechnol*. 2024; doi: 10.1038/s41565‐024‐01676‐4). This device can autonomously and selectively activate the display of cytotoxic ligand patterns in tumor microenvironments. The study demonstrated the feasibility and potential for developing DNA origami that can selectively trigger cytotoxicity to combat cancer.

**Kathryn E. Meier**, from Washington State University in Spokane, presented a study using a global proteomics approach to gain a broader perspective on the role of CCN1 in lysophosphatidic acid (LPA)‐mediated signaling. She utilized the human prostate cancer cell line PC‐3 as a model system, which was incubated with LPA, in the absence and presence of CCN1 knockdown. Kathryn Meier discussed how this study revealed a significant number of proteins whose expression was either increased or decreased by LPA. Importantly, the knockdown of CCN1 altered LPA's ability to increase levels of multiple proteins involved in cell‐substrate adhesion and signaling, such as the metastasis‐associated gene in colon cancer 1 (MACC1) and thrombospondin‐1 (THBS1). This study demonstrates that global proteomics is a promising approach to identify new CCN signaling pathways and potentially uncover therapeutic targets associated with modified signaling in human prostate cancer and other diseases.

**Gary Fisher** from the University of Michigan Medical School highlighted the important function of MMP‐1 in accelerating dermal aging and tumor formation. He discussed that MMP‐1 is a matrix metalloproteinase predominantly expressed in dermal fibroblasts with the capacity to cleave collagen fibrils that serve as substrates for other members of the MMP family. To address the molecular mechanism in more detail, he generated a mouse model of dermal aging by expressing human MMP1 in fibroblasts. He showed that at 5 months of age, the respective mice exhibited significant histological alterations that closely resembled those seen in aged human skin. Importantly, the mice further displayed a strikingly increased susceptibility to carcinoma driven by the HRas oncogene as assessed in a tri‐transgenic model. This model, in addition to the MMP‐1 transgene, expressed a tamoxifen‐activated oncogenic mutant of HRas. These findings clearly indicate that MMP‐1 is crucial in dermal aging and skin tumor formation and that these processes are not strictly cell‐autonomous.

## 5 CONCLUSION

The scientific quality and audience of the 12th workshop on the CCN family of genes provided one of the most exciting times for the ICCNS. The standard of the presentations and their discussions confirmed once more that the expansion of the session topics introduced in 2022 at the Nice 11th CCN workshop is truly beneficial. Indeed the collaborative era is alive and should attract other members of the factors superfamilies that are also representative of the Cellular Communication Networks, of which CCN1–6 are only a small group of biologically important members. Discussions undertaken in April 2022 have now been refined at the 12th workshop and the ARBIOCOM is presently on track to the launching of the first World Conference on Cell Signaling and Communication (See Event Communication in this issue).

The new Steering Committee chaired in Oslo by Milos Marinkovic will have to work on proposals leading to the organization and governance establishment of a multi‐faceted ARBIOCOM project. This should lead shortly to the organization of delegate and executive Committee elections, providing the framework of ARBIOCOM activities, representing all of the constituent societies who expressed interest in joining this project. A website is in progress and should become available soon. In the meantime, all questions regarding ARBIOCOM can be addressed to Annick or Bernard Perbal at info@arbiocom.org. Aside from these exploratory aspects, the 12th CCN workshop was the place for very sound communications, as summarized above.

However, as already discussed and presented by Bernard Perbal in his scientific introduction to the meeting, there are “black holes” whose significance should really attract young investigators and need to be tackled, either by the CCN community members and/or via the collaborations fostered by the ARBIOCOM. Among them, the detection of nuclear CCN proteins, once reduced to a few anecdotal examples, rapidly became a generalized observation that stands alone, while the biological significance of the nuclear addressing increased in highly proliferative normal and tumoral cells is extremely puzzling and might “hide” a central regulatory signaling realm whose manifestations had previously been reported by the previous work of Prochiantz. It may be at the center of regulatory processes using the signaling vesicles that are legion in the biological fluids (see review by Alain Prochiantz and Ariel A. Di Nardo, *Methods Mol Biol*. 2022; 2383:33–44).

Presentations in this workshop put again “on the grill” the questions relating to the relative abundance of the different CCN proteins in target tissues. Behind this CCN “abundance” aspect, stands the question of their tissue specific expression in developing and differentiating tissues, a key component of the spatio‐temporal regulation of CCN activities and interactions with a wealth of other regulatory factors whose expression is also tightly controlled. Another issue that needs to be resolved is the extent to which CCN proteins are recognized by specific receptors on the plasma membrane that are responsible for transmembrane signaling and activation of intracellular signaling pathways. Tackling these coordinated pathways is the basis of balanced multifactorial dependent growth control and differentiation both in normal and pathological conditions, as pointed out in a few communications during our meeting. The mastering of CCN proteins acting in concert, either as biological antagonist or agonist, remains to be understood at molecular comprehensive levels permitted by the development of powerful spatial biological approaches, which are already applied to personalized medical approaches.

Finally, presentations in this workshop also stressed at different levels, where precise and thorough 3D structure‐function approaches were required to shed a brighter light on the biological functions that are unique and/or shared by other members of the family. Whether this must be the future of CCN research, it will tightly depend upon the combinatorial application of such technologies to address questions that are still standing aside in spite of their fundamental interest for the best of humanity.

As a final conclusion, the excitement of the audience was a solid proof that the CCN field is developing in the right direction and should attract more external participants for the next 2026 CCN workshop to be held in Chicago by Joon‐Il Jun who responded very positively to our invitation.

## AUTHOR CONTRIBUTIONS

Håvard Attramadal, Ralf Weiskirchen, and Bernard Perbal have participated in the conceptualization, writing, and editing of the manuscript.

## CONFLICT OF INTEREST STATEMENT

Bernard Perbal is President of the CCN Society, a non‐profit association and the Editor‐in‐Chief of the Journal of Cell Communication and Signaling (JCCS), the official journal of the International CCN Society. Ralf Weiskirchen and Håvard Attramadal are Executive Editors of JCCS. The authors declare that they have no known competing financial interests or personal relationships that could have appeared to influence the work reported in this paper.

## ETHICS STATEMENT

Not applicable.

## Data Availability

Research data are not shared.

